# *Notes from the Field:* Suspected Medetomidine Withdrawal Syndrome Among Fentanyl-Exposed Patients — Philadelphia, Pennsylvania, September 2024–January 2025

**DOI:** 10.15585/mmwr.mm7415a2

**Published:** 2025-05-01

**Authors:** Samantha Huo, Kory London, Lauren Murphy, Emily Casey, Philip Durney, Maya Arora, Rita McKeever, Abriana Tasillo, Dennis Goodstein, Brendan Hart, Jeanmarie Perrone

**Affiliations:** ^1^Department of Emergency Medicine, Perelman School of Medicine, University of Pennsylvania, Philadelphia, Pennsylvania; ^2^Center for Addiction Medicine and Policy, Perelman School of Medicine, University of Pennsylvania, Philadelphia, Pennsylvania; ^3^Department of Emergency Medicine, Thomas Jefferson University, Philadelphia, Pennsylvania; ^4^Department of Emergency Medicine, Lewis Katz School of Medicine, Temple University, Philadelphia, Pennsylvania; ^5^Philadelphia Poison Control Center, Philadelphia, Pennsylvania; ^6^Department of Pharmacy, Hospital of the University of Pennsylvania, Philadelphia, Pennsylvania; ^7^Department of Medicine, Thomas Jefferson University, Philadelphia, Pennsylvania; ^8^Department of Pharmacy, Thomas Jefferson University Hospital, Philadelphia, Pennsylvania.

**Summary**

**What is already known about this topic?**

Medetomidine, a nonopioid sedative not approved for use in humans, replaced xylazine as the most common drug adulterant in the Philadelphia, Pennsylvania, illegal opioid supply during the last 4 months of 2024.

**What is added by this report?**

During September 2024–January 2025, 165 patients at three Philadelphia health systems were hospitalized for fentanyl withdrawal complicated by profound autonomic dysfunction, including severe hypertension and tachycardia. This syndrome was resistant to medications that had previously been effective in managing fentanyl and xylazine withdrawal but was responsive to dexmedetomidine.

**What are the implications for public health practice?**

Health care providers and public health agencies should be aware of shifts in the drug supply over time that might change patient signs and symptoms. The findings in this report indicate that medetomidine withdrawal syndrome is life-threatening and can require a substantial escalation in care compared with the typical opioid and xylazine withdrawal syndromes. Public health agencies should consider testing for medetomidine in their regional drug supplies.

Medetomidine, a synthetic alpha-2 adrenoreceptor agonist, is a new drug adulterant that was detected in 72% of illegal opioid samples tested in Philadelphia, Pennsylvania, during the last 4 months of 2024. During the same period, detection of xylazine (previously the most common adulterant) decreased from 98% to 31% of samples (*1*), and health care providers at hospitals in Philadelphia noticed an increasing number of hospitalized patients with a severe drug withdrawal syndrome distinct from fentanyl and xylazine withdrawal, characterized by profound autonomic dysfunction, such as severe hypertension and tachycardia. This report aims to increase awareness of the presence of medetomidine in the illegal opioid supply, characterize the emerging medetomidine withdrawal syndrome, and describe measures to provide effective patient care for this life-threatening syndrome.

## Investigation and Outcomes

During fall 2024, in response to emerging awareness of a newly recognized medetomidine withdrawal syndrome, addiction medicine and medical toxicology faculty members at three Philadelphia health systems (health systems A, B, and C) began maintaining a list of patients identified with the syndrome, including those they had helped care for or provided consultation for, as well as patients referred by other health care providers. The faculty members reviewed electronic health records of patients who were admitted to the three health systems during September 1, 2024–January 31, 2025, and whose withdrawal syndrome was characterized by severe signs and symptoms that were not resolved by established treatment protocols for fentanyl and xylazine withdrawal. Overall, 165 patients were identified who demonstrated one or more signs or symptoms such as agitation, anxiety, severe hypertension, tachycardia, tremor without clonus or hyperreflexivity, and vomiting, resistant to increasing doses of opioids (e.g., fentanyl, hydromorphone, methadone, or oxycodone), sedatives (e.g., diazepam, droperidol, haloperidol, lorazepam, midazolam, phenobarbital, or propofol), and adjunctive opioid and xylazine withdrawal medications (clonidine, ketamine, olanzapine, ondansetron, or tizanidine) (*2*). Median age was 38 years (IQR = 33–43 years). This evaluation was reviewed and approved by the institutional review boards of health systems A, B, and C.

Among the 165 patients, 150 (91%) required intensive care unit (ICU) care, including 39 (24%) who received endotracheal intubation (Table). A total of 137 (83%) patients were treated with and responded to dexmedetomidine* infusion, a drug eventually recognized as potentially effective; medetomidine is an enantiomer^†^ of dexmedetomidine, and prolonged dexmedetomidine exposure can induce a withdrawal syndrome manageable with controlled weaning from the drug. Traditional dosages of dexmedetomidine (0.2–1.5 *μ*g/kg/hr) (*3*) were used and titrated to control symptoms or sedate patients with intubation. In a majority of patients requiring dexmedetomidine, the drug was titrated to a maximum dosage of 1.5 *μ*g/kg/hr. Duration of infusion varied, depending on the patient. Use of oral alpha-2 agonists, such as clonidine, was limited because of vomiting. Patients were also treated with antihypertensive medications titrated to blood pressure levels ≤180/120 mm Hg. Complications secondary to severe hypertension or tachycardia included altered mental status with computed tomography (CT)- or magnetic resonance imaging (MRI)-documented posterior reversible encephalopathy syndrome^§^ in three patients, and non-ST elevation myocardial infarction (NSTEMI) secondary to demand ischemia (insufficient blood supply to meet the heart’s oxygen demand) with positive high-sensitivity troponin, indicating potential damage to the heart muscle in a substantial number of patients^¶^. Findings of severe withdrawal syndromes typically associated with other sedatives (alcohol, barbiturate, or benzodiazepine), such as seizures or hallucinations, were infrequent. Routine testing of specimens from all 165 patients by hospital laboratories confirmed universal fentanyl exposure. Testing for medetomidine or its metabolites using liquid phase chromatography with mass spectrometry was available at health system A; all 55 patients treated at health system A received a positive test result for 3-hydroxy medetomidine.

**TABLE:** Characteristics of patients hospitalized with combined opioid and suspected medetomidine withdrawal syndrome — three health systems, Philadelphia, Pennsylvania, September 2024–January 2025

Characteristic	No. (%)			
	Health system A (n = 55)	Health system B (n = 48)	Health system C (n = 62)	Total (N = 165)
**Age, yrs, median (IQR)**	37 (33–45)	38 (35–41)	38 (32–45)	**38 (33–43)**
**Sex**				
Female	12 (22)	20 (42)	17 (27)	**49 (30)**
Male	43 (78)	28 (58)	45 (73)	**116 (70)**
Race and ethnicity*				
Black or African American, non-Hispanic	6 (11)	6 (13)	15 (24)	**27 (16)**
White, non-Hispanic	44 (80)	34 (71)	25 (40)	**103 (62)**
Hispanic or Latino	5 (9)	0 (—)	18 (29)	**23 (14)**
Other	0 (—)	8 (17)	4 (7)	**12 (7)**
**Clinical findings and hospital course**				
Maximum heart rate (beats per minute), median (IQR)	144 (125–155)	136 (118–156)	148 (140–157)	**145 (132–156)**
Maximum systolic blood pressure (mm Hg), median (IQR)	191 (172–211)	196 (171–224)	200 (185–215)	**195 (175–215)**
Maximum diastolic blood pressure (mm Hg), median (IQR)	111 (103–123)	127 (109–137)	131 (119–143)	**122 (109–136)**
Treated with dexmedetomidine	51 (93)	35 (73)	51 (82)	**137 (83)**
Intubation/Mechanical ventilation	12 (22)	11 (23)	16 (26)	**39 (24)**
Admitted to intensive care unit	49 (89)	44 (92)	57 (92)	**150 (91)**
**Disposition**				
Home	15 (27)	28 (58)	32 (52)	**75 (45)**
Patient-directed discharge	14 (26)	13 (27)	25 (40)	**52 (32)**
Residential drug treatment	14 (26)	7 (15)	0 (—)	**21 (13)**
Law enforcement custody	12 (22)	0 (—)	5 (8)	**17 (10)**

* Persons of Hispanic or Latino (Hispanic) origin might be of any race but are categorized as Hispanic; all racial groups are non-Hispanic.

## Preliminary Conclusions and Actions

The syndrome described in this report is similar to that described among ICU patients with days-long exposure to dexmedetomidine, an enantiomer of medetomidine, who experience an autonomic withdrawal syndrome with vomiting and agitation when dexmedetomidine is discontinued (*4*,*5*). In the patients described in this report, these signs and symptoms were not resolved by increasing doses of medications previously effective in managing fentanyl and xylazine withdrawal; however, they were responsive to dexmedetomidine, as described in the management of dexmedetomidine withdrawal (*4*,*5*). Health care providers and public health agencies need to be aware of this life-threatening withdrawal syndrome because it can require substantial escalations in care compared with the typical opioid and xylazine withdrawal syndromes. Public health agencies should consider testing for medetomidine in their regional drug supplies.

## Footnotes

* Dexmedetomidine is an alpha-2 agonist medication that is used for sedation in an ICU and operating room.

† Enantiomer molecules are mirror images of each other and are not superimposable (e.g., right and left hands).

§ Posterior reversible encephalopathy syndrome is a neurologic disorder characterized by brain swelling that can arise when blood pressure is severely increased. The syndrome is diagnosed by cross-sectional brain imaging such as CT or MRI scan.

¶ Only health system C collected these data, although the patient population at health systems A and B were similar to that of health system C. NSTEMI was defined as a positive high-sensitivity troponin. Of health system C’s 62 patients, 39 patients had an NSTEMI, and 13 had a normal or negative high-sensitivity troponin test result. Ten patients were not tested for high-sensitivity troponin.
