# *Erratum*: Vol. 74, No. 15

**DOI:** 10.15585/mmwr.mm7417a3

**Published:** 2025-05-15

The report “*Notes from the Field*: Suspected Medetomidine Withdrawal Syndrome Among Fentanyl-Exposed Patients — Philadelphia, Pennsylvania, September 2024–January 2025” contained an error. 

On page 268, reference 1 in the References should have read, “**PA Groundhogs. Correction: PAG releases new adulterant report. Philadelphia, PA: PA Groundhogs; 2025.  **https://pagroundhogs.org/news/f/pag-releases-new-adulterant-report.”

